# Development and Feasibility of a Yoga-Based Motor Assessment Tool (Y-MAT) with AI-Enabled Scoring for Children with ADHD

**DOI:** 10.1192/j.eurpsy.2026.10557

**Published:** 2026-07-31

**Authors:** V. P, B. Holla, H. Bhargav, E. Sharma, R. Naidu L

**Affiliations:** 1 Department of Integrative Medicine; 2 Department of Child and Adolescent Psychiatry, NIMHANS; 3 Centre for Development of Advanced Computing, Bangalore, India

## Abstract

**Introduction:**

Motor dysfunction in ADHD is increasingly recognized as a core manifestation of neurodevelopmental disruption rather than an incidental problem. Yet there is no consensus gold-standard motor assessment, and most tools emphasize broad milestones rather than neuromuscular dysregulation.

**Objectives:**

Aim: Develop a Yoga-Based Motor Assessment Tool (Y-MAT) for children with ADHD.

Primary objectives: Develop and validate the tool; pilot test feasibility.

Secondary objective: Explore artificial intelligence and video-analytics for digitizing motor assessment.

**Methods:**

Seven motor domains were identified through review of literature and mapped to Yoga-tasks through expert consensus (n = 6). The Y-MAT was administered to 11 children with ADHD and 10 controls by a trained psychiatrist, video-recorded, and scored by 2 blinded human raters. AI-based scoring was also implemented using Google’s MediaPipe and YOLOv8 for pose recognition and movement analysis. All children were also assessed using the ADHD Rating Scale (ADHD-RS) and the Vineland Social Maturity Scale (VSMS) to evaluate symptom severity and social functioning. Feasibility, Inter-rater reliability (Cohen’s kappa) and Concurrent Validity was measured.

**Results:**

The Y-MAT demonstrated high acceptability, engagement and a minimal dropout rate.The ADHD scores show a moderate negative correlation with both ratings, with slightly stronger associations for AI-based scoring, indicating that greater ADHD severity was associated with poorer neuromuscular regulation. Inter-rater reliability ranged from fair to moderate agreement, with Impulse Control of Vrikshasana (p = 0.0001) and Motor Sequencing of Surya Namaskar (p = 0.0032) exhibiting the strongest and statistically significant agreement. AI-based scores moderately correlated with human ratings.

**Table 1.** Selected Yoga-tasks mapped to their corresponding motor domains Table 1. Long description.

YOGA TASK	MOTOR DOMAINS
A. Dynamic Trikonasana/ Triangle pose	Body Awareness & Spatial Orientation (A1), Bilateral & Cross Limb Coordination (A2)
B. Vrikshasana/ Tree pose	Static & Dynamic Balance (B1), Impulse Control & Movement Regulation (B2)
C. Surya Namaskar/ Sun Salutation	Motor Sequencing & Rhythm (C1), Bilateral & Cross Limb Coordination (C2), Body Awareness & Spatial Orientation (C3), Static & Dynamic Balance (C4)
D. Alternating Mudras	Fine Motor Dexterity & Control (D1), Bilateral & Cross Limb Coordination (D2)
E. Bhramari	Breath Control (E1), Impulse Control & Movement Regulation (E2)

**Conclusions:**

The Y-MAT is a feasible and engaging approach to assess motor dysfunction relevant to ADHD. Preliminary evidence supports concurrent validity and the promise of AI-assisted scoring for scalable, real-time, objective evaluation.

**Disclosure of Interest:**

None Declared

## Long descriptions

### Table 1. Long description

The table consists of two columns titled YOGA TASK and MOTOR DOMAINS.

Row A. Dynamic Trikonasana or Triangle pose maps to Body Awareness and Spatial Orientation (A 1) and Bilateral and Cross Limb Coordination (A 2).

Row B. Vrikshasana or Tree pose maps to Static and Dynamic Balance (B 1) and Impulse Control and Movement Regulation (B 2).

Row C. Surya Namaskar or Sun Salutation maps to Motor Sequencing and Rhythm (C 1), Bilateral and Cross Limb Coordination (C 2), Body Awareness and Spatial Orientation (C 3), and Static and Dynamic Balance (C 4).

Row D. Alternating Mudras maps to Fine Motor Dexterity and Control (D 1) and Bilateral and Cross Limb Coordination (D 2).

Row E. Bhramari maps to Breath Control (E 1) and Impulse Control and Movement Regulation (E 2).

Navigate back to Table 1..

